# Prevalence and associated factors of suicidal behaviors among Bangladeshi rural community people: Findings from the ‘BD ComMen Study’

**DOI:** 10.1371/journal.pone.0279271

**Published:** 2022-12-20

**Authors:** Mohammed A. Mamun, Firoj Al-Mamun, Johurul Islam, Mohammad Muhit

**Affiliations:** 1 CHINTA Research Bangladesh, Savar, Dhaka, Bangladesh; 2 Department of Public Health & Informatics, Jahangirnagar University, Savar, Dhaka, Bangladesh; 3 Department of Public Health, University of South Asia, Dhaka, Bangladesh; 4 CSF Global, Banani, Dhaka, Bangladesh; Bangladesh University of Professionals, BANGLADESH

## Abstract

**Background:**

Suicide is considered as one of the major public health concerns, which can be prevented with cost-effective and timely intervention. In Bangladesh, very few studies assessed the suicidal behavior of rural community people. Thus, this Bangladesh Community Mental Health Study (**BD ComMen Study**) attempted to understand the current situation of suicidality in Bangladeshi rural community people considering three-time frames: lifetime, past year, and past month.

**Methods:**

A cross-sectional study was conducted in a rural community in Bangladesh between May 17 and 31, 2022, using a cluster sampling technique. Information on socio-demographics, COVID-19-related factors, depression, anxiety, insomnia, and suicidal behaviors was collected. The Chi-square test or Fisher’s exact test and logistic regression were used to analyze the data.

**Results:**

During their lifetime, 33.1% of the rural community people had suicidal thoughts, whereas 5.5% made a plan for suicide and 1.8% attempted suicide. The prevalence of past-year suicidal ideation was 3.9%, whereas 1.4% had a suicide plan. In addition, 0.6% had past-month suicidal thoughts, although none of them had planned or attempted suicide. The factors associated with suicidal behaviors included males, lower age, lower educational grade, low-earning jobs, living in a government-provided house, family history of mental health and suicide, and suffering from anxiety and insomnia.

**Conclusions:**

Suicidal behaviors among the rural community people are of great concern as most of the rural people in Bangladesh do not have enough mental health literacy for treatment-seeking due to a high level of mental health-related stigma. Thus, this study would likely help to initiate further studies and stimulate suicide prevention programs, because most suicide can be prevented.

## 1 Introduction

Suicide is the intentional death by injuring oneself. The World Health Organization reported that over 700,000 annual deaths occur due to suicide, of which 77% of suicides are from low- and middle-income countries like Bangladesh as per data from 2019 [1]. In Bangladesh, 7.8 age-standardized suicide per 100,000 people accounting for approximately 10,000 suicide each year, was reported in 2012 [2]. The recent World Health Organization report suggested that the rate was 3.85 in 2020, accounting for about six thousand annual suicide deaths in the country and ranked 153^rd^ worldwide [3]. However, it is claimed that the COVID-19 pandemic worsened the situation in Bangladesh, reporting an increasing trend of suicide [4–8].

For every suicide completion, people have to pass through several stages called suicidal behaviors. Suicidal behaviors include suicidal ideation or thought, a suicide plan, and a suicide attempt. The World Health Organization estimated that a prior suicide attempt in the general population is the most important suicide risk factor for suicide [1]. For instance, on average, a successful suicide completion is followed by 18 prior suicide attempts, as per the World Health Organization (cited in [9]).

In Bangladesh, there is a lack of studies conducted on suicidal behaviors, especially amongst people residing in rural areas [10]. Though several studies were conducted during the COVID-19 pandemic [4–8], none were conducted specifically on rural community people. However, very few studies were conducted in the country, all of which are decades older, except Begum et al.’s study [11], which was conducted in 2013. For instance, from the Health and Demographic Surveillance System of the icddr,b, a total of 625 elderly aged 60 to 95 years from the rural Matlab areas were studied to identify the prevalence of depression and suicidal ideation. About 23% of suicidal ideation over the past two weeks was found in the study from 2003 to 2004, which was prevalent among women (28% vs. 17%) [12]. Another community-based study from the same area, Matlab, conducted in 2005, reported that among the antenatally depressed participants at 34–35 weeks of pregnancy, 14% had self-harm thoughts [13]. Further, within 2706 ever-married women of 15–49 years, 11% to 14% and 5% to 6% lifetime and past month suicidal ideation rates, respectively, were found in a study conducted in 2001 [14].

The aforementioned studies that were conducted in rural areas were among (i) elderly [12], (ii) pregnant women [13], (iii) ever-married women [14], and adolescents aged 14 to 19 years [11]; and all of which are approximately a decade-older. Thus, there is a knowledge gap on suicidal behaviors related information among people in rural adult communities. Therefore, this study conducted a community-based cluster survey to identify the prevalence and factors of suicidal behaviors in Bangladeshi rural community people. The findings of this study are anticipated to create baseline data for suicidal behaviors in the rural community that can help to conduct further studies and prompt suicide preventive actions.

## 2 Methods

### 2.1 Study procedure and participants

This cross-sectional household survey was conducted from the Bangladesh Community Mental Health Study (**BD ComMen Study**) project among the rural people of Bangladesh. A team of trained research assistants implemented the data collection from 17 to 31 May, 2022. For data collection, a face-to-face interview was conducted at the home of the participants. About 585 responses were collected from the field, and 490 were kept for the formal analysis after removing the incomplete responses. Participants were included in the analysis if they were aged between 18 and 59 years, whereas those not participating willingly were excluded from the study.

### 2.2 Sampling frame

The Pabna district’s Bera Upazila was specifically chosen for this study. One of the nine unions in Bera Upazila was chosen randomly to include participants. The selected union is subdivided into 9 wards. From there, two wards were selected using the lottery method. Therefore, the two wards, were regarded as cluster units and adult participants (aged between 18 and 59) from those two wards were recruited for this study.

### 2.3 Sample size calculation

The sample size was calculated using the following formula

$$n=z^{2}pq/d^{2}$$

Where, n = sample size, z = 1.96 for 95% of the confidence interval, p = prevalence, 5% [11]; q = (1-p); d = 5% margin of error. In this way, the estimated sample size was 73. After adjusting for sampling error with a 10% non-response rate, the estimated sample size was 80 for each cluster. Therefore, there need to be at least (80*2 = 160) samples. The present study comprised 490 participants, which showed an adequate sample size.

### 2.4 Measures

#### 2.4.1 Sociodemographic factors

This survey collected information related to age, gender, marital status, educational qualification, monthly family income, occupation, having any debt, house type, presence of any chronic condition, family history of mental health, and family history of suicide. Monthly family income was categorized as low income: Less than 15000 Bangladeshi Taka (BDT) ≈ less than $160.68; middle income: 15000 to 30000 BDT ≈ $160.68 to $321.37; and high income: more than 30000 BDT ≈ more than $321.37. BDT from USD was converted using the current money exchange rate.

#### 2.4.2 COVID-19 related factors

The COVID-19-related factors included being infected themselves and being infected and dying their friends or family members due to COVID-19. Responses were collected based on binary (yes/no) responses.

#### 2.4.3 Depression

Depression was screened using the two-item Patient Health Questionnaire-2 [15]. The scale consisted of a 4-point Likert scale (0 = not at all, to 3 = nearly every day) with a score ranging from 0 to 6. A cut-off score of ≥3 indicated depression among the participants. The Cronbach’s alpha was 0.76 in the present study.

#### 2.4.4 Anxiety

Anxiety was screened using the two-item Generalized Anxiety Disorder-2 [16]. The responses were recorded based on a 4-point Likert scale (0 = not at all, to 3 = nearly every day) with a score ranging from 0 to 6. A cut-off score of ≥3 indicated anxiety among the participants. The Cronbach’s alpha was 0.76 in the present study.

#### 2.4.5 Insomnia

Insomnia was assessed using the two-item Insomnia Severity Index [17]. The responses were collected based on a 5-point Likert scale (0 = very satisfied to 4 = very dissatisfied) with a score ranging from 0 to 8. A cut-off score of ≥6 detects the presence of insomnia. The Cronbach’s alpha was 0.78 in the present study.

#### 2.4.6 Suicidal behaviors

To assess suicidal behavior, the participants were asked three questions about three types of suicidal behaviors: lifetime, past year, and past month. First, for suicidal ideation, the participants were asked if they had thought of committing suicide, whereas if they planned for suicide, and if they attempted suicide. These questions were followed by the concept of suicidality and the measures used to assess suicidal behaviors by the previous pieces of literature [12, 18, 19].

### 2.5 Ethics

The study was approved by the University of South Asia, Dhaka, Bangladesh. Additionally, the Helsinki Declaration guidelines were also followed. Informed consent was taken from the interviewees after describing the nature and study purpose and then the interview was started. For the participants with no education, verbal consent was collected and the research team signed on their behalf of them after making them aware of it, and this was approved by the ethics committee. No monetary or non-monetary remuneration was offered for participating in the study. They were ensured of the confidentiality of their records and had the right to withdraw their participation at any time during the interview.

### 2.6 Statistical analysis

Data analysis was conducted using the IBM SPSS (Statistical Package for Social Science) software version 25. Descriptive statistics (i.e., frequency and percentages) and inferential statistics (Chi-square and Fisher exact test) were carried out to analyze the data. Association between the variables was estimated using either the Chi-square test or Fisher’s exact test. Fisher exact test was used when more than 20% of cells have <5 expected frequencies. Binary logistic regression analysis was conducted to examine the associated factors with the outcome variables. A *p*-value <0.05 was set as statistical significance with a 95% confidence interval.

## 3 Results

### 3.1 Characteristics of the participants

The mean age of the participants was 36.97 (±10.56) years. About 42% belonged to the 31–44 years of age group, 53.5% were females, 88.8% were married, 42.9% had no formal education, 47.8% were housewives, and 84.7% had up to 15000 monthly family income. In addition, 91.2% lived in their own house, 64.3% reported having family debt, 26.3% had a chronic disease, 13.3% had a family mental health history, and 2.9% had a family history of suicide. Furthermore, 1.4% had an infection with the COVID-19 virus, 4.9% had a family history of COVID-19 infection, and 1.2% reported the death of family members due to COVID-19 (Table 1).

**Table 1:** Characteristics of the participants.

Study variables	Total (n, %)
**Socio-demographic information**	
**Age group**	
18–30 years	143; 29.2%
31–44 years	206; 42%
45–59 years	118; 24.1%
**Gender**	
Male	228; 46.5%
Female	262; 53.5%
**Marital status**	
Married	435; 88.8%
Unmarried	31; 6.3%
Divorcee/separated/widow	22; 4.5%
**Educational status**	
No formal education	210; 42.9%
Primary level	174; 35.5%
Secondary level	60; 12.2%
Higher Secondary level	31; 6.3%
Bachelor and above	13; 2.7%
**Occupation**	
Farmer	73; 14.9%
Day labor	66; 13.5%
Businessman	67; 13.7%
Housewife	234; 47.8%
Teacher	6; 1.2%
Employed	6; 1.2%
Student	27; 5.5%
Others	9; 1.8%
**Monthly income (BDT)**	
Up to 15000	415; 84.7%
15001 to 30000	60; 12.2%
More than 30000	10; 2%
**House type**	
Government provided	36; 7.3%
Own	447; 91.2%
**Family debt**	
Yes	315; 64.3%
No	173; 35.3%
**Chronic disease**	
Yes	129; 26.3%
No	358; 73.1%
**Family mental health history**	
Yes	65; 13.3%
No	423; 86.3%
**Family suicide history**	
Yes	14; 2.9%
No	475; 96.9%
**COVID-19 related information**	
**COVID-19 status**	
Yes	7; 1.4%
No	469; 95.7%
Don’t know or did not test	12; 2.4%
**Family history of COVID-19**	
Yes	24; 4.9%
No	464; 94.7%
**Family history of COVID-19 death**	
Yes	6; 1.2%
No	480; 98%

### 3.2 Prevalence of suicidal behaviors

**Fig 1:**
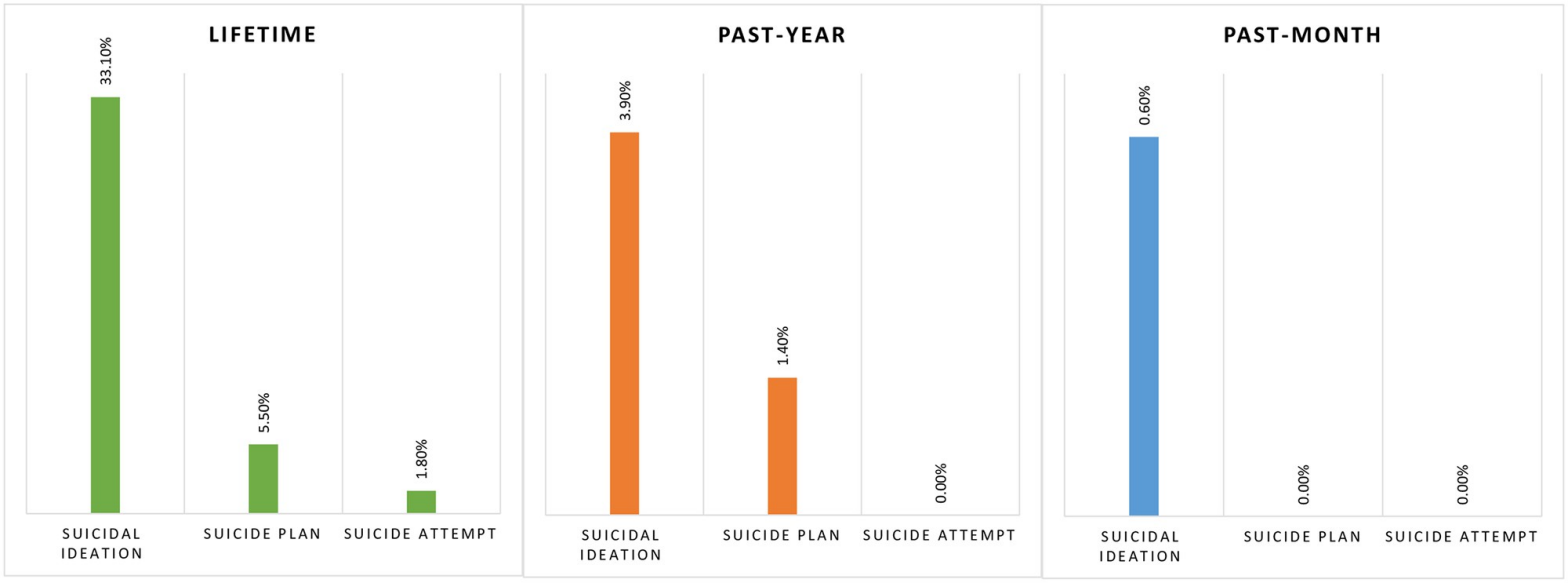
Prevalence of suicidal behaviors among Bangladeshi rural people.

The prevalence of suicidal behaviors is presented in Fig 1. Of the participants, 33.1% had reported thinking of suicide during their lifetime, whereas 5.5% made a plan for suicide and 1.8% attempted suicide during their lifetime. The prevalence of past-year suicidal ideation was 3.9%, whereas 1.4% had a suicide plan. In addition, 0.6% had past-month suicidal thoughts, although none of them had planned or attempted suicide.

### 3.3 Associations with *lifetime* suicidal behaviors

Table 2 presents the associations between study variables and lifetime suicidal behaviors. The results suggested that age group (χ^2^ = 10.111, *p* = 0.006), educational status (χ^2^ = 11.325, *p* = 0.023), occupation (χ^2^ = 14.152, *p* = 0.040), house type (χ^2^ = 26.840, *p* <0.001), family debt (χ^2^ = 10.901, *p* = 0.001), suffering from chronic disease (χ^2^ = 5.840, *p* = 0.016), COVID-19 infection status (χ^2^ = 7.379, *p* = 0.021), anxiety (χ^2^ = 4.404, *p* = 0.036), and insomnia (χ^2^ = 6.643, *p* = 0.010) were significantly associated with lifetime suicide ideation. Whereas, gender (χ^2^ = 3.430, *p* = 0.011), marital status (χ^2^ = 9.393, *p* = 0.006), educational status (χ^2^ = 14.109, *p* = 0.004), occupation (χ^2^ = 18.281, *p* = 0.005), house type (χ^2^ = 14.099, *p* = 0.002), suffering from chronic disease (χ^2^ = 15.694, *p* <0.001), family mental health history (χ^2^ = 9.873, *p* = 0.005), family history of COVID-19 infection (χ^2^ = 8.909, *p* = 0.039) were significantly associated with lifetime suicide plan. In addition, suffering from chronic disease (χ^2^ = 7.602, *p* = 0.013) were the only associated factors of lifetime suicide attempt (Table 3).

**Table 2:** Association between the study variables and *lifetime* suicidal behaviors.

Variables	Suicide ideation (n = 162; 33.1%)			Suicide plan (n = 27; 5.5%)			Suicide attempt (n = 9; 1.8%)		
	Yes; *n* (%)	χ^2^ test value	*p*-value	Yes; *n* (%)	χ^2^ test value	*p*-value	Yes; *n* (%)	χ^2^ test value	*p*-value
***Socio-demographics***									
**Age group**									
18–30 years	37; 24.3%	10.111	0.006	3; 11.1%	5.522	0.063	-	4.603 ^a^	0.083
31–44 years	83; 54.6%			14; 51.9%			6; 66.7%		
45–59 years	32; 21.1%			10; 37%			3; 33.3%		
**Gender**									
Male	85; 52.5%	3.430	0.064	19; 70.4%	6.474	0.011	6; 66.7%	1.494	0.222
Female	77; 47.5%			8; 29.6%			3; 33.3%		
**Marital status**									
Married	137; 85.6%	3.781	0.151	20; 74.1%	9.393 ^a^	0.006	8; 88.9%	1.562 ^a^	0.418
Unmarried	12; 7.5%			2; 7.4%			-		
Divorcee/separated/widow	11; 6.9%			5; 18.5%			1; 11.1%		
**Educational status**									
No formal education	85; 53.1%	11.325	0.023	20; 74.1%	14.109 ^a^	0.004	8; 88.9%	5.567 ^a^	0.160
Primary level	44; 27.5%			2; 7.4%			1; 11.1%		
Secondary level	20; 12.5%			4; 14.8%			-		
Higher secondary level	8; 5%			1; 3.7%			-		
Bachelor and above	3; 1.9%			-			-		
**Occupation**									
Farmer	34; 21.3%	14.152^a^	0.040	7; 25.9%	18.281 ^a^	0.005	2; 22.2%	8.017 ^a^	0.254
Day labor	27; 16.9%			10; 37%			4; 44.4%		
Businessman	14; 8.8%			-			-		
Housewife	73; 45.6%			8; 29.6%			3; 33.3%		
Teacher	1; 0.6%			-			-		
Employed	1; 0.6%			-			-		
Student	8; 5%			2; 7.4%			-		
Others	2; 1.3%			-			-		
**Monthly family income (BDT)**									
Up to $160.68	140; 87%	0.377	0.828	26; 96.3%	1.804 ^a^	0.352	9; 100%	0.926 ^a^	0.678
$160.68 to $321.37	18; 11.2%			1; 3.7%			-		
More than $321.37	3; 1.9%			-			-		
**House type**									
Government provided	26; 16.3%	26.840	<0.001	7; 25.9%	14.099 ^a^	0.002	1; 11.1%	0.178 ^a^	0.505
Own	134; 83.8%			20; 74.1%			8; 88.9%		
**Family debt**									
Yes	121; 74.7%	10.901	0.001	22; 81.5%	3.609	0.057	6; 66.7%	0.587 ^a^	1.00
No	41; 25.3%			5; 18.5%			3; 33.3%		
**Chronic disease**									
Yes	54; 33.3%	5.840	0.016	16; 59.3%	15.694	<0.001	6; 66.7%	7.602 ^a^	0.013
No	108; 66.7%			11; 40.7%			3; 33.3%		
**Family mental health history**									
Yes	24; 14.8%	0.470	0.493	9; 33.3%	9.873 ^a^	0.005	3; 33.3%	3.181 ^a^	0.105
No	138; 85.2%			18; 66.7%			6; 66.7%		
**Family suicide history**									
Yes	4; 2.5%	0.135	0.713	2; 7.4%	2.113 ^a^	0.178	-	0.270 ^a^	1.00
No	158; 97.5%			25; 92.6%			9; 100%		
**COVID-19 related information**									
**COVID-19 infection status**									
Yes	3; 1.9%	7.379^a^	0.021	1; 3.7%	1.741 ^a^	0.426	-	0.677 ^a^	1.00
No	158; 98.1%			26; 96.3%			9; 100%		
Don’t know or did not test	-			-			-		
**Family history of COVID-19 infection**									
Yes	5; 3.1%	1.688	0.194	-	1.482 ^a^	0.387	-	0.474 ^a^	1.00
No	156; 96.9%			27; 100%			9; 100%		
**Family history of COVID-19 death**									
Yes	3; 1.9%	0.781^a^	0.403	2; 7.4%	8.909 ^a^	0.039	1; 11.1%	7.336 ^a^	0.107
No	158; 98.1%			25; 92.6%			8; 88.9%		
**Mental health related information**									
**Depression**									
Yes	19; 11.7%	0.062	0.804	5; 18.5%	1514 ^a^	0.212	2; 22.2%	1.113 ^a^	0.267
No	143; 88.3%			22; 81.5%			7; 77.8%		
**Anxiety**									
Yes	27; 16.7%	4.404	0.036	6; 22.2%	2.630 ^a^	0.125	1; 11.1%	0.011 ^a^	1.00
No	135; 83.3%			21; 77.8%			8; 88.9%		
**Insomnia**									
Yes	11; 6.8%	6.643	0.010	2; 7.4%	1.119 ^a^	0.261	1; 11.1%	1.433 ^a^	0.288
No	151; 93.2%			25; 92.6%			8; 88.9%		

Note:

^a^ Fisher’s exact test.

**Table 3:** Association between the study variables and *past-year* suicidal behaviors.

Variables	Suicide ideation (n = 19; 3.9%)			Suicide plan (n = 7; 1.4%)		
	Yes; *n* (%)	χ^2^ test value	*p*-value	Yes; *n* (%)	χ^2^ test value	*p*-value
***Socio-demographics***						
**Age group**						
18–30 years	1; 5.6%	6.713	0.035	-	3.333	0.152 ^a^
31–44 years	9; 50%			3; 50%		
45–59 years	8; 44.4%			3; 50%		
**Gender**						
Male	14; 73.7%	5.858	0.016	5; 71.4%	1.769	0.259 ^a^
Female	5; 26.3%			2; 28.6%		
**Marital status**						
Married	12; 66.7%	18.047 ^a^	<0.001	3; 50%	12.266	0.003 ^a^
Unmarried	-			-		
Divorcee/separated/widow	6; 33.3%			3; 50%		
**Educational status**						
No formal education	15; 83.3%	13.056 ^a^	0.006	5; 83.3%	2.828	0.498 ^a^
Primary level	2; 11.1%			1; 16.7%		
Secondary level	-			-		
Higher secondary level	-					
Bachelor and above	1; 5.6%			-		
**Occupation**						
Farmer	4; 22.2%	17.678 ^a^	0.006	1; 16.7%	7.579	0.352 ^a^
Day labor	8; 44.4%			3; 50%		
Businessman	-			-		
Housewife	5; 27.8%			2; 33.3%		
Teacher	-			-		
Employed	1; 5.6%			-		
Student	-			-		
Others	-			-		
**Monthly family income (BDT)**						
Up to $160.68	18; 947%	0.578 ^a^	0.660	7; 100%	0.786	0.658 ^a^
$160.68 to $321.37	1; 5.3%			-		
More than $321.37	-			-		
**House type**						
Government provided	8; 42.1%	34.429 ^a^	<0.001	3; 42.9%	12.907	0.011 ^a^
Own	11; 57.9%			4; 57.1%		
**Family debt**						
Yes	16; 84.2%	3.340	0.068	7; 100%	3.900	0.055 ^a^
No	3; 15.8%			-		
**Chronic disease**						
Yes	15; 78.9%	27.942	<0.001	6; 85.7%	12.794	0.002 ^a^
No	4; 21.1%			1; 14.3%		
**Family mental health history**						
Yes	12; 63.2%	42.532^a^	<0.001	3; 42.9%	5.367	0.053 ^a^
No	7; 36.8%			4; 57.1%		
**Family suicide history**						
Yes	2; 10.5%	4.174 ^a^	0.099	-	0.209	1.00 ^a^
No	17; 89.5%			7; 100%		
**COVID-19 related information**						
**COVID-19 status**						
Yes	1; 5.6%	2.619 ^a^	0.286	1; 14.3%	5.502	0.107 ^a^
No	17; 94.4%			6; 85.7%		
Don’t know or did not test	-			-		
**Family history of COVID-19**						
Yes	1; 5.6%	0.016 ^a^	0.603	-	0.367	1.00 ^a^
No	17; 94.4%			7; 100%		
**Family history of COVID-19 death**						
Yes	1; 5.6%	2.862 ^a^	0.204	-	0.089	1.00 ^a^
No	17; 94.4%			7; 100%		
**Mental health related information**						
**Depression**						
Yes	3; 15.8%	0.413 ^a^	0.461	2; 28.6%	2.145	0.180 ^a^
No	16; 84.2%			5; 71.4%		
**Anxiety**						
Yes	4; 21.1%	1.427 ^a^	0.273	1; 14.3%	0.028	1.00 ^a^
No	15; 78.9%			6; 85.7%		
**Insomnia**						
Yes	2; 10.5%	2.623 ^a^	0.151	1; 14.3%	2.260	0.232 ^a^
No	17; 89.5%			6; 85.7%		

### 3.4 Associations with *past-year* suicidal behaviors

Table 3 presents the associations between the study variables and past-year suicidal behaviors. The results suggested that age group (χ^2^ = 6.713, *p* = 0.035), gender (χ^2^ = 5.858, *p* = 0.005), marital status (χ^2^ = 18.047, *p* <0.001), educational status (χ^2^ = 13.056, *p* = 0.006), occupation (χ^2^ = 17.678, *p* = 0.006), house type (χ^2^ = 34.429, *p* <0.001), suffering from chronic disease (χ^2^ = 27.942, *p* <0.001), and family mental health history (χ^2^ = 42.532, *p* <0.001) were significantly associated with past-year suicide ideation. In addition, marital status (χ^2^ = 12.266, *p* = 0.003), house type (χ^2^ = 12.907, *p* = 0.011), family debt (χ^2^ = 3.900, *p* = 0.055), suffering from chronic disease (χ^2^ = 12.794, *p* = 0.002), family mental health history (χ^2^ = 5.367, *p* = 0.053) were significantly associated with past-year suicide plan (Table 3).

### 3.5 Associations with *past-month* suicidal behavior

Table 4 presents the associations between the study variables and past-month suicide ideation. The results suggested that marital status (χ^2^ = 10.620, *p* = 0.006), house type (χ^2^ = 15.344, *p* = 0.015), suffering from chronic disease (χ^2^ = 8.377, *p* = 0.018), family mental health history (χ^2^ = 7.441, *p* = 0.048), and COVID-19 infection status (χ^2^ = 8.571, *p* = 0.044) were significantly associated with past-year suicide ideation (Table 4).

**Table 4:** Association between the study variables and *past-month* suicide ideation and its associated factors.

Variables	Suicide ideation (n = 3; 0.6%)			Risk factors	
	Yes; *n* (%)	χ^2^ test value	*p*-value	Odds ratio (95% CI)	*p-*value
**Socio-demographics**					
**Age group**					
18–30 years	-	2.464	0.258 ^a^		
31–44 years	1; 33.3%				
45–59 years	2; 66.7%				
**Gender**					
Male	2; 66.7%	0.492	0.600 ^a^	2.31 (0.20–25.64)	0.495
Female	1; 33.3%			1	
**Marital status**					
Married	1; 33.3%	10.620	0.006 ^a^		
Unmarried	-				
Divorcee/separated/widow	2; 66.7%				
**Educational status**					
No formal education	3; 100%	3.706	0.524 ^a^		
Primary level	-			-	
Secondary level	-			-	
Higher secondary level	-			-	
Bachelor and above	-				
**Occupation**					
Farmer	1; 33.3%	7.469	0.539 ^a^		
Day labor	1; 33.3%			-	
Businessman	-			-	
Housewife					
Teacher	-			-	
Employed	-			-	
Student	-			-	
Others	-				
**Monthly family income (BDT)**					
Up to $160.68	3; 100%	1.204	1.00 ^a^		
$160.68 to $321.37	-				
More than $321.37	-				
**House type**					
Government provided	2; 66.7%	15.344	0.015 ^a^	26.23 (2.32–296.71)	0.008
Own	1; 33.3%			1	
**Family debt**					
Yes	3; 100%	1.658	0.556 ^a^		
No	-				
**Chronic disease**					
Yes	3; 100%	8.377	0.018 ^a^		
No	-				
**Family mental health history**					
Yes	2; 66.7%	7.441	0.048 ^a^	13.39 (1.19–149.90)	0.035
No	1; 33.3%			1	
**Family suicide history**					
Yes	-	0.089	1.00 ^a^		
No	3; 100%				
**COVID-19 related information**					
**COVID-19 status**					
Yes	1; 33.3%	8.571	0.044 ^a^		
No	2; 66.7%				
Don’t know or did not test	-				
**Family history of COVID-19**					
Yes	-	0.156	1.00 ^a^		
No	2; 100%				
**Family history of COVID-19 death**					
Yes	-	0.038	1.00 ^a^		
No	3; 100%				
**Mental health related information**					
**Depression**					
Yes	1; 33.3%	1.481	0.301 ^a^	4.00 (0.35–44.95)	0.260
No	2; 66.7%			1	
**Anxiety**					
Yes	1; 33.3%	1.249	0.325 ^a^	3.62 (0.32–40.62)	0.296
No	2; 66.7%			1	
**Insomnia**					
Yes	1; 33.3%	7.504	0.106 ^a^	13.82 (1.19–159.99)	0.036
No	2; 66.7%			1	

^a^ Fisher’s exact test; Age group, marital status, education, occupation, monthly family income, family debt, chronic disease, family suicide history, COVID-19 status, family history of COVID-19, and family history of COVID-19 death were not included in the model due to multicollinearity.

### 3.6 Factors associated with *lifetime* suicidal behaviors

Table 5 presents the factors associated with lifetime suicide behaviors. The results suggested that 31–44 years age group was more likely to have suicide ideation (OR = 1.81, 95% CI = 1.10–2.96, *p* = 0.007), and age group 18–30 years have less likely suicide ideation (OR = 0.93, 95% CI = 0.54–1.62, *p* = 0.007) compared with age group 45–59 years. In addition, having no formal education (OR = 2.26, 95% CI = 0.60–8.47, *p* = 0.025), primary level (OR = 1.12, 95% CI = 0.29–4.28, *p* = 0.025), secondary level (OR = 1.66, 95% CI = 0.41–6.74, *p* = 0.025), and higher secondary level (OR = 1.15, 95% CI = 0.25–5.30, *p* = 0.025) were more likely to have suicide ideation than who had bachelor and above education. Higher risk of suicide ideation was reported among the farmers (OR = 3.05, 95% CI = 0.59–15.68, *p* = 0.045), day labor (OR = 2.42, 95% CI = 0.46–12.57, *p* = 0.045), housewife (OR = 1.58, 95% CI = 0.32–7.82, *p* = 0.045), and student (OR = 1.47, 95% CI = 0.25–8.69, *p* = 0.045), whereas teacher (OR = 0.70, 95% CI = 0.04–10.01, *p* = 0.045), and employed person (OR = 0.70, 95% CI = 0.04–10.01, *p* = 0.045) were at lower risk compared to other group of people. Additionally, living in a government provided house (OR = 6.07, 95% CI = 2.84–12.94, *p* < 0.001), having family debt (OR = 2.0, 95% CI = 1.32–3.04, *p* = 0.001), suffering from chronic disease (OR = 1.66, 95% CI = 1.09–2.52, *p* = 0.016), anxiety (OR = 1.78, 95% CI = 1.03–3.09, *p* = 0.038), and depression (OR = 3.34, 95% CI = 1.27–8.78, *p* = 0.015) were at higher risk of lifetime suicide ideation than their counterparts.

**Table 5:** Factors associated with *lifetime* suicide behaviors.

Variables	Suicide ideation		Suicide plan		Suicide attempt	
	Odds ratio (95% CI)	*p*-value	Odds ratio (95% CI)	*p*-value	Odds ratio (95% CI)	*p*-value
**Socio-demographics**						
**Age group**						
18–30 years	0.93 (0.54–1.62)	0.007	0.23 (0.06–0.86)	0.089		
31–44 years	1.81 (1.10–2.96)		0.78 (0.33–1.83)		-	
45–59 years	1		1			
**Gender**						
Male	1.42 (0.97–2.08)	0.064	2.88 (1.23–6.72)	0.014	2.33 (0.57–9.43)	0.235
Female	1		1		1	
**Marital status**						
Married	0.46 (0.19–1.08)	0.161	0.16 (0.05–0.48)	0.005		
Unmarried	0.63 (0.20–1.90)		0.23 (0.04–1.34)			
Divorcee/separated/widow			1			
**Educational status**						
No formal education	2.26 (0.60–8.47)	0.025				
Primary level	1.12 (0.29–4.28)					
Secondary level	1.66 (0.41–6.74)					
Higher secondary level	1.15 (0.25–5.30)					
Bachelor and above	1					
**Occupation**						
Farmer	3.05 (0.59–15.68)	0.045				
Day labor	2.42 (0.46–12.57)					
Businessman	0.92 (0.17–4.95)					
Housewife	1.58 (0.32–7.82)					
Teacher	0.70 (0.04–10.01)					
Employed	0.70 (0.04–10.01)					
Student	1.47 (0.25–8.69)					
Others	1					
**Monthly family income (BDT)**						
Up to $160.68	1.18 (0.30–4.66)	0.829				
$160.68 to $321.37	1.00 (0.23–4.31)					
More than $321.37	1					
**House type**						
Government provided	6.07 (2.84–12.94)	<0.001	5.15 (2.01–13.18)	**0.001**	1.56 (0.19–12.89)	0.676
Own	1		1		1	
**Family debt**						
Yes	2.00 (1.32–3.04)	0.001	2.52 (0.93–6.78)	0.067	1.10 (0.27–4.45)	0.893
No	1		1		1	
**Chronic disease**						
Yes	1.66 (1.09–2.52)	0.016	4.46 (2.01–9.90)	**<0.001**	5.77 (1.42–23.43)	0.014
No	1		1		1	
**Family mental health history**						
Yes	1.20 (0.70–2.08)	0.494	3.61 (1.54–8.44)	**0.003**	3.36 (0.82–13.79)	0.092
No	1		1		1	
**Family suicide history**						
Yes	0.80 (0.24–2.59)	0.714	3.00 (0.63–14.13)	0.165	-	-
No	1		1		1	
**COVID-19 related information**						
**Family history of COVID-19**						
Yes	0.52 (0.19–1.41)	0.20				
No	1		1			
**Family history of COVID-19 death**						
Yes	2.03 (0.40–10.21)	0.387	9.10 (1.59–52.07)	0.013	11.80 (1.23–112.84)	0.032
No			1		1	
**Mental health related information**						
**Depression**						
Yes	1.07 (0.59–1.94)	0.804	1.87 (0.68–5.17)	0.224	2.30 (0.46–11.39)	0.305
No	1		1		1	
**Anxiety**						
Yes	1.78 (1.03–3.09)	0.038	0.46 (0.17–1.19)	0.111	1.11 (0.13–9.10)	0.917
No	1		1		1	
**Insomnia**						
Yes	3.34 (1.27–8.78)	0.015	0.44 (0.09–2.05)	0.301	3.41 (0.40–28.83)	0.260
No	1		1		1	

For Lifetime suicide ideation: COVID-19 status was not included in the model due to multicollinearity.

For the Lifetime suicide plan: education, occupation, monthly family income, COVID-19 status, and family history of COVID-19 status were not included in the model due to multicollinearity.

For insomnia: Age group, marital status, education, occupation, monthly family income, family suicide history, and family history of COVID-19 were not included in the model due to multicollinearity.

Male was at higher risk of lifetime suicide plan (OR = 2.88, 95% CI = 1.23–6.72, p = 0.014) than females. Married (OR = 0.16, 95% CI = 0.05–0.48, p = 0.005), and unmarried (OR = 0.23, 95% CI = 0.04–1.34, p = 0.005) women were less likely to have suicide plan than divorced/separated/widow women. Additionally, living in a government provided house (OR = 5.15, 95% CI = 2.01–13.18, p = 0.001), suffering from chronic disease (OR = 4.46, 95% CI = 2.01–9.90, p <0.001), having a family mental health history (OR = 3.61, 95% CI = 1.54–8.44, p = 0.003), and family history of COVID-19 death (OR = 9.10, 95% CI = 159–52.07, p = 0.013) were at higher risk of planning suicide than those who were not.

Participants suffering from chronic disease (OR = 5.77, 95% CI = 1.42–23.43, p = 0.014) and having a family history of COVID-19 death (OR = 11.80, 95% CI = 1.23–112.84, p = 0.032) were at higher risk of attempting lifetime suicide than those who were not.

### 3.7 Factors associated with *past-year* suicidal behaviors

Table 6 shows the factors associated with past-year suicide behaviors. The results suggested that male was at a higher risk of past-year of suicide ideation (OR = 3.36, 95% CI = 1.19–9.48, p = 0.022) than females. Additionally, living in a government provided house (OR = 11.32, 95% CI = 4.21–30.40, p <0.001), suffering from chronic disease (OR = 11.64, 95% CI = 3.78–35.79, p <0.001), and having a family mental health history (OR = 13.45, 95% CI = 5.07–35.67, p <0.001) were at a higher risk of suicide ideation than those who were not.

**Table 6:** Factors associated with *past-year* suicide behavior.

Variables	Suicide ideation		Suicide plan	
	Odds ratio (95% CI)	*p*-value	Odds ratio (95% CI)	*p*-value
**Socio-demographics**				
**Age group**				
18–30 years	0.09 (0.01–0.78)	0.085		
31–44 years	0.62 (0.23–1.67)			
45–59 years	1			
**Gender**				
Male	3.36 (1.19–9.48)	0.022	2.91 (0.56–15.17)	0.204
Female	1		1	
**House type**				
Government provided	11.32 (4.21–30.40)	<0.001	10.06 (2.16–46.87)	0.003
Own	1		1	
**Family debt**				
Yes	3.03 (0.87–10.55)	0.081		
No	1		1	
**Chronic disease**				
Yes	11.64 (3.78–35.79)	<0.001	17.41 (2.07–146.08)	0.008
No	1		1	
**Family mental health history**				
Yes	13.45 (5.07–35.67)	<0.001	5.06 (1.10–23.18)	0.036
No	1		1	
**Family suicide history**				
Yes	4.49 (0.93–21.65)	0.061		
No	1			
**COVID-19 related information**				
**Family history of COVID-19**				
Yes	1.14 (0.14–8.96)	0.899		
No				
**Family history of COVID-19 death**				
Yes	5.44 (0.60–49.20)	0.131		
No			1	
**Mental health related information**				
**Depression**				
Yes	1.51 (0.42–5.36)	0.523	3.24 (0.61–17.14)	0.166
No	1		1	
**Anxiety**				
Yes	1.97 (0.63–6.16)	0.241	1.19 (0.14–10.12)	0.868
No	1		1	
**Insomnia**				
Yes	3.34 (0.71–15.72)	0.126	4.56 (0.52–40.07)	0.170
No	1		1	

For suicide ideation: Marital status, education, occupation, monthly family income, and COVID-19 status were not included in the model due to multicollinearity.

For suicide plan: Age group, marital status, education, occupation, monthly family income, family debt, family suicide history, COVID-19 status, family history of COVID-19, and family history of COVID-19 death were not included in the model due to multicollinearity.

Living in a government provided house (OR = 10.06, 95% CI = 2.16–46.87, p = 0.003), suffering from chronic disease (OR = 17.41, 95% CI = 2.07–146.08, p = 0.008), and having a family mental health history (OR = 5.06, 95% CI = 1.10–23.18, p = 0.036) were at higher risk of planning suicide than those who were not (Table 6).

### 3.8 Factors associated with *past-month* suicidal behaviors

Table 4 presents the factors associated with the past month suicide ideation. Results suggested that participants living in the government-provided house were at 26 times higher risk of past month suicide ideation (OR = 26.23, 95% CI = 2.32–296.71, *p* = 0.008) than those who lived in their own house. Additionally, having a family mental health history was approximately 13 times higher risk of past month suicide ideation (OR = 13.39, 95% CI = 1.19–149.90, *p* = 0.035).

## 4 Discussion

The present study investigated the prevalence and associated factors of suicidal behaviors in the rural community people in Bangladesh. Results suggested that about one-third of the participants (33.1%) thought of committing suicide in their lifetime, whereas 5.5% and 1.8% had the prevalence of lifetime suicide plans and suicide attempts, respectively. In the past year, 3.9% had reported suicidal thoughts, and 1.4% had planned for suicide. Lastly, the prevalence of suicidal ideation for the past month was 0.6%. Previous Bangladeshi studies conducted in rural areas found heterogeneous prevalence rates. For instance, 23% of suicidal ideation over the past two weeks was reported among the elderly [12], whereas the rate was 11% to 14% and 5% to 6% for lifetime and past-month suicidal ideation, respectively, among ever-married women [14], and lifetime suicidal ideation was 5% among adolescent in rural Bangladesh [11]. However, adult participants from city areas reported the prevalence of six-month suicidal ideation and suicide plan as 5.8% and 3.4%, respectively [20]. After the COVID-19 pandemic, a systematic review identified that the prevalence rate of suicidal ideation ranges from 5% to 19.0%, whereas those studies conducted earlier in the pandemic, had a lower prevalence rate because of the prevalence estimation timeframe being short [8]. No studies have been conducted among the rural community people during the pandemic; hence, this study’s prevalence rates appear a little bit higher than the online survey studies conducted immediately after the pandemic’s inception.

Gender is considered as an important factor in suicide and suicidal behaviors. Globally, males are less likely to have suicidal behaviors but more likely to commit suicide [21]. For instance, the World Health Organization reported 13.7 suicide deaths per 100,000 population for males, while the rate was 7.5 for females [22]. A Korean study found that suicide attempts were 42.7% among females, whereas it was 18.1% among males [23]. However, it has been claimed that suicide trends are opposite in Bangladesh, with a higher suicide rate among females [24]. In this study, there was no significant difference in the gender reporting lifetime suicidal ideation and suicide attempt; although the males had 2.88 times higher risk of a lifetime suicide plan. Considering the past-year suicidal ideation, males were at 3.36 times higher risk, but other suicidal behaviors were insignificant as per gender status. However, given the gender-based finding of this study, it appears that males are at higher risk of suicidality in Bangladeshi rural communities and they should be prioritized for early prevention by identifying the potential factors influencing their higher risk of suicidality.

Age and education status were significantly associated with lifetime suicidal thoughts. That is, the lower age group and having lower education status were more likely to report a higher suicidal ideation rate. In addition, as per the occupation status and lifetime suicidal ideation relationship, farmers, and day-labor groups were at higher risk of suicidality in this study. This may be because those groups of people have lower education and are the most low-paid occupational. It is worth mentioning that this study also finds that people with lower education are at greater risk of suicidal ideation. Those with lower earnings are more likely to adopt loans for their livelihood. Sometimes the scheme of loan repaying and its associated interest may lead burden on their life. Many suicides in Bangladesh have been reported due to poverty and associated familiar financial deficits. For instance, poverty has been cited as the second most suicide-affecting factor accounting for 24.3% of suicide death casualties [25]. Similarly, unemployment and financial distress at the time of the COVID-19 pandemic appeared as the prominent risk factors for suicide in Bangladeshi people [8]. Those living below the poverty line are less likely to have their habitat and enough support from their belonging society, leading them to have shelter in government-provided houses. In this study, it was found that those people living in government-provided houses were at a six and five-time higher risk of lifetime suicidal ideation and suicide plan, respectively. Similarly, ten and eleven times more likely to report past-year suicidal ideation and suicide plan.

Family history of mental disorders and suicide have been well-established as the risk factors for suicide and suicidal behaviors. Moreover, the risk of mental suffering is elevated among offspring; for instance, higher anxiety disorders are reported in these parents with bipolar disorder, depression, schizophrenia, etc. [26], as well as suicidal behaviors of the youths from their parents with positive history [27]. However, this study found no significant relationship between a family history of mental health and lifetime suicidal behaviors (except for a lifetime suicide plan). But this was a significant factor of the past-year suicidal ideation and suicide plan, and the past month’s suicide ideation. Thus, people from mentally vulnerable families should be enlisted in the priority of suicide prevention groups.

Suicidal behaviors largely depend on the factors like personal well-being. For instance, any people’s medical conditions and psychological status would determine how prone the person will be to suicide. Historical literature suggests that individuals suffering from chronic diseases are more likely to have a higher risk of mental disorders and suicidality [28, 29]. A 4.46- and 5.77-times more elevated risk of lifetime suicide plan and attempt, respectively, were found among the participants with chronic medical conditions, whereas the risk appears 11.64 and 17.41 times higher for past-year suicidal ideation and suicide plan, respectively. Moreover, it has been said that about 90% of suicide deaths occur due to mental health-related issues, whereas depression and mood disorders are reported as the main culprit of suicide [30, 31]. In this study, all the assessed mental health problems were not the risk factors for suicidal behaviors across three different times. For instance, depressed participants in this study were not at a greater risk of suicidal behaviors. But anxious and insomniac participants were at higher risk of reporting lifetime suicidal ideation. However, such unusual findings should be further studied with more rigorous tools to explore the association between mental health problems and suicidal behaviors in the rural community people of the country.

## 5 Strength and limitations

Two points demand to be acknowledged that may weaken the study, that is, (i) the nature of the study: a cross-sectional study, (ii) methodological issues: social desirability bias while conducting the face-to-face survey, and memory recall bias for responding to suicidal behaviors. However, after a decade, this study adds some findings on suicidal behaviors in the rural community people of Bangladesh. This study’s findings are anticipated to create baseline data regarding the rural community people’s suicidal behaviors that can help to conduct further studies. In addition, this is the first study to identify several associated factors of suicidal behaviors considering different time frames such as lifetime, past year, and past month considering this cohort.

## 6 Conclusions

Suicidal behaviors among the rural community are of great concern as most of the rural people in Bangladesh do not have enough literacy for treatment-seeking and are highly stigmatized. In this study, one-third of the participants had lifetime suicidal ideation (33.1%), whereas about 4% and less than one percent had past-year and past-month suicidal ideation, respectively. Since suicide can be prevented, it is high time to start suicide prevention programs considering the vulnerable cohort of rural communities. In addition, suicide prevention programs such as increasing mental health literacy, counseling people suffering from mental health problems and chronic condition, and taking special care for people living in government-provided houses is recommended for rural community people.

## Supporting information

S1 Data ComMen data. (SAV)
